# The prioritization and development of key social and structural indicators to address gaps in a framework for monitoring the Strategies toward Ending Preventable Maternal Mortality (EPMM): Results of an iterative expert technical consultation

**DOI:** 10.7189/jogh.11.04057

**Published:** 2021-10-09

**Authors:** R Rima Jolivet, Malia Skjefte, Jewel Gausman, Ana Langer

**Affiliations:** Improving Maternal Health Measurement Capacity & Use Project; Women & Health Initiative; Department of Global Health & Population; Harvard T.H. Chan School of Public Health, Boston, Massachusetts, USA

## Abstract

**Background:**

Since 2014, iterative technical work has captured stakeholder demand and channeled it toward improving maternal health measurement, to support SDG 3.1. Strategies toward Ending Preventable Maternal Mortality (EPMM) (2015) turned a broad lens on upstream systemic determinants of maternal health and survival highlighted in 11 Key Themes. A monitoring framework was developed to help countries track progress across these domains. This process yielded requests for additional indicators where stakeholders identified gaps for tracking EPMM Key Themes. In response, two technical consultations aimed at affirming the measurement gaps, specifying the constructs for measurement, and fully elaborating the metadata to allow them to be monitored.

**Methods:**

Measures for development were prioritized based on multi-stakeholder dialogues in five countries, and data collected from government officials and UN partners in twenty countries on perceived need for proposed additional indicators. Sixty-one participants representing expertise in measure development and the topical areas covered took part across both consultations. Measures were developed through two simultaneous participatory online consultations stratified by focus area, comprising videos, discussion forums, polls, and live Zoom meetings.

**Results:**

Eight candidate indicators relevant to priority recommendations in the EPMM Strategies are presented. Each includes a definition, numerator and denominator (if applicable), method of estimation, disaggregation factors, preferred data source(s), and expected periodicity. Four address gaps in measures of fundamental rights-related determinants of maternal health at national and subnational level, including women’s reproductive autonomy; participative accountability for maternal health outcomes; and Respectful Maternity Care. Four strengthen the ability to count, track, and link births and maternal deaths and causes of death.

**Conclusions:**

The proposed indicators correspond to specific EPMM Key Themes, filling gaps identified by multiple stakeholders, and respond to calls for a broadened approach to measurement and for indicators that track the social and health-systems determinants of maternal health. They reflect inputs and aspirations of numerous stakeholders, gathered over time and across various platforms. The iterative, discursive exploration of the concepts for measurement and the need for metrics to track them responds to recent calls for measure development to be carried out in more inclusive ways, and to be primarily concept- and user-driven.

The broad lens of the Sustainable Development Goals (SDGs) prioritizes integrated, coordinated, and multi-sectoral approaches to the health of individuals, societies, and the planet. The UN Secretary General’s updated Global Strategy for Women’s, Children’s and Adolescents’ Health (2016-2030) [1] goes beyond survival to include efforts to ensure that women, families, and communities can thrive and transform to meet their full potential.

As countries move along the pathway of the obstetric transition and progress toward ending preventable maternal mortality, the causes of maternal death are also widening. Along with indirect causes of death, the more distal social and structural determinants of maternal health and survival are taking on greater significance. To understand and address the full range of causes of death, high-quality, nationally representative data on deaths are needed; however, at the beginning of the SDGs in 2015, no deaths data were available in seventy-three countries due to lack of functional civil registration and vital statistics systems [2].

The Strategies toward Ending Preventable Maternal Mortality (EPMM) (EPMM Strategies) [3], released in 2015 by the World Health Organization to provide a strategic framework for maternal health under the SDGs, likewise turns a broad lens on the determinants of maternal health and survival, both direct, and distal - including social, political, economic, and systemic determinants - highlighted in “11 Key Themes” (**Table 1**).

**Table 1:** EPMM Key Themes

Guiding principles	1. Empower women, girls, families and communities
2. Integrate maternal and newborn health, protect and support the mother-baby dyad
3. Prioritize country ownership, leadership, and supportive legal, regulatory and financial frameworks
4. Apply a human-rights framework to ensure that high-quality reproductive, maternal, and newborn health care is available, accessible and acceptable to all who need it
**Cross-cutting actions**	5. Improve metrics, measurement systems, and data quality
	6. Prioritize adequate resources and effective health care financing
**Five strategic objectives**	7. Address inequities in access to and quality of sexual, reproductive, maternal and newborn health care
	8. Ensure universal health coverage for comprehensive sexual, reproductive, maternal, and newborn health care
	9. Address all causes of maternal mortality, reproductive and maternal morbidities and related disabilities
	10. Strengthen health systems to respond to the needs and priorities of women and girls
	11. Ensure accountability in order to improve quality of care and equity

EPMM – Ending Preventable Maternal Mortality

The EPMM Strategies present priority recommendations in eleven domains reflecting the full spectrum of determinants of maternal health and survival; these “11 Key Themes” emerged through consultation with global and national stakeholders through a number of channels, including an official member state consultation and public call for comments hosted by the WHO. In line with the SDG agenda, the EPMM Strategies go beyond the health sector and cover structural and societal factors that influence maternal health outcomes including chance of death. Such a multi-sectoral lens is particularly important for maternal health, where outcomes are more dependent on other sectors and upstream determinants than other more “vertical” domains of health.

The increasing complexity of the current era calls for improved approaches to monitoring maternal and newborn health. We need to broaden the range of what is monitored, at the same time that we apply more precision in choosing measures that are fit for purpose and worth the burden of collection. The principle of parsimony calls for focused attention to what matters, which varies from differing perspectives, in different contexts, and for different uses. At the advent of the SDG era, the so-called “Kirkland commentary” [4] identified five principles for maternal and newborn health measurement: *focus* through core indicator sets, *relevance* of measures to end-users, measurement *innovation*, inclusion of *equity* measures, and supportive global *leadership*. A review of these principles at the five-year mark resulted in the call for a sixth, overarching principle of *country ownership* through which the first five principles should be filtered [5]. Additionally, there were calls for increased focus on policy and health system-level drivers in health measurement [6], as measurement efforts have traditionally focused quite narrowly on health services and interventions, neglecting measures of societal determinants that empower people to achieve full health, or systems determinants that create an enabling environment for effective service delivery and quality of care.

To enable countries to track progress toward achieving the priority recommendation in each domain highlighted in the 11 Key Themes, work was undertaken to develop a comprehensive monitoring framework for the EPMM Strategies, in two phases, described elsewhere [7,8]. Whereas Phase I focused on reaching consensus on a minimum set of core maternal health coverage and outcome measures for cross-country comparisons, Phase II focused specifically on identifying indicators to address the distal causes (social, political, and economic determinants) of maternal mortality, by seeking to answer the question, “What are the 1-3 strongest available indicators for monitoring each EPMM Key Theme?” From 2016-2017, multiple rounds of modified Delphi process were implemented to develop a menu of indicators suitable for national/sub-national monitoring. Indicators were selected by stakeholders based on pre-specified criteria focused on relevance to the EPMM thematic area, importance across a range of contexts, interpretability and utility to end users, as well as validity, feasibility and harmonization with other global measure development initiatives. A secondary outcome of this process was the emergence of a set of requests for additional indicators linked to each EPMM Key Theme, reflecting instances when participants in the process identified a need for new metrics to fill measurement gaps. Stakeholder feedback suggested that developing a small number of additional indicators would be an important contribution to a comprehensive monitoring framework for the EPMM Strategies. In total, twenty-nine additional indicators were requested to fill such gaps (**Table 2**).

**Table 2:** Additional indicators requested by EPMM Phase II stakeholders

EPMM key Theme	Requested ndicator	Health system strengthening	Human rights	Universal health coverage	Empowering women & girls	Improving metrics & measurement
1	Proportion of women aged 15-49 who make their own informed and empowered decisions regarding sexual relations, contraceptive use, and reproductive health care, and the timing and number of births		Yes		Yes	
1	Number of district local governments that articulate efforts of sectors accredited in its geographic area and monitor results in each community	Yes				
2	Availability of services for mothers and newborns that are provided in the same setting	Yes				
2	Presence of national information system(s) that are able to record and report data as described by ICD-PM, linking outcomes (births and deaths) to maternal and perinatal conditions, and to report annually on characteristics of births, deaths, and other vital events to produce statistics relevant to monitoring of reproductive health and mortality					Yes
3	Country holds routine national health sector reviews with basic criteria for broad stakeholder participation, including a structured process to engage political and financial decision makers		Yes			
4	Presence of Respectful Maternity Care (RMC) as a right in the national health plan(s)		Yes			
4	Presence of a component that specifically addresses the Universal Rights of Childbearing Women (RMC Charter) in the national pre-service education curriculum for all midwifery service providers		Yes			
4	Percentage of health care facilities in a country that offer a minimum package of sexual and reproductive health services			Yes		
4	Proportion of received complaints on the right to health investigated and adjudicated by a national human rights institution, ombudsperson, or other mechanism AND the proportion of these responded to effectively by the government		Yes			
4	Whether the right to health is currently justiciable and enforceable under the law and subject to investigation by national accountability mechanism(s)		Yes			
4	Presence of a national strategy and action plan with budget allocations on sexual and reproductive health which is periodically reviewed and monitored through participatory processes and disaggregated by prohibited ground of discrimination (per ESCR General Comment No. 22 (2016) on the right to sexual and reproductive health)		Yes			
5	Percentage of health workers using MNCAH data for decision-making					Yes
5	Death and birth registration coverage					Yes
5	Annual reporting based on a set of national indicators that are harmonized with global targets to inform annual health sector reviews and other planning cycles	Yes				
6	Types of financing mechanisms for the delivery of maternal health goods and/or services identified, tested, and officially adopted	Yes				
7	Percentage of eligible population covered by national social protection programs			Yes		
7	Presence of a national policy/strategy to ensure engagement of civil society organization representatives in national level planning of sexual, reproductive, maternal, newborn, child, and adolescent health programs		Yes		Yes	
8	Presence of a national, defined minimum benefits package for sexual, reproductive, maternal, and newborn health, as recommended by the Midwifery Services Framework of the International Confederation of Midwives		Yes	Yes		
8	Composite Coverage Indicator			Yes		
8	Share of the population that are not pushed into poverty due to health care expenditures			Yes		
9	Maternal near miss ratio					Yes
9	Percentage of health facilities with a water source or water supply in or near (within 500m) the facility for use for drinking, personal hygiene, medical activities, cleaning, laundry, cooking and a power source	Yes				
10	Availability of functional routine care: obstetric and newborn care facilities	Yes				
10	Percentage of facilities that demonstrate readiness to deliver specific services: family planning, antenatal care, basic emergency obstetric care, and newborn care INCLUDING: functioning emergency transport; life-saving commodities for maternal and newborn health; and a water source or supply in or near (within 500m) the facility for use for drinking, personal hygiene, medical activities, cleaning, laundry, and cooking	Yes				
10	Evidence that maternal and newborn health policies, strategies, and plans of action were formulated in coordination with other sectors	Yes				
11	The maternal death surveillance and response system is reviewed annually in terms of completeness of surveillance and quality of the response, including actions to improve quality of care	Yes				
11	Presence of a national grievance mechanism (ex: ombudsman) to receive and facilitate resolution of concerns and grievances from project-affected parties related to [SRMNCAH]		Yes			
11	The national RMNCAH strategy/plan of action mandates community participation in decision-making, delivery of health services, and monitoring and evaluation				Yes	
11	Presence of a national policy/strategy to ensure engagement of civil society organization representatives in periodic review of national programs for sexual, reproductive, maternal, newborn, child, and adolescent health (SRMNCAH)		Yes		Yes	

EPMM – Ending Preventable Maternal Mortality

In 2017, the Women and Health Initiative (W&HI) at the Harvard T.H. Chan School of Public Health launched the Improving Maternal Health Measurement Capacity and Use project (IMHM Project), aimed at strengthening metrics and measurement capacity to support country efforts to drive improvement in maternal health and survival. The full body of work aimed at capturing stakeholder demand and channeling it into activities and resources to improve maternal health measurement as a core strategy for ending preventable maternal deaths is displayed in a Theory of Change diagram (**Figure 1**).

**Figure 1:**
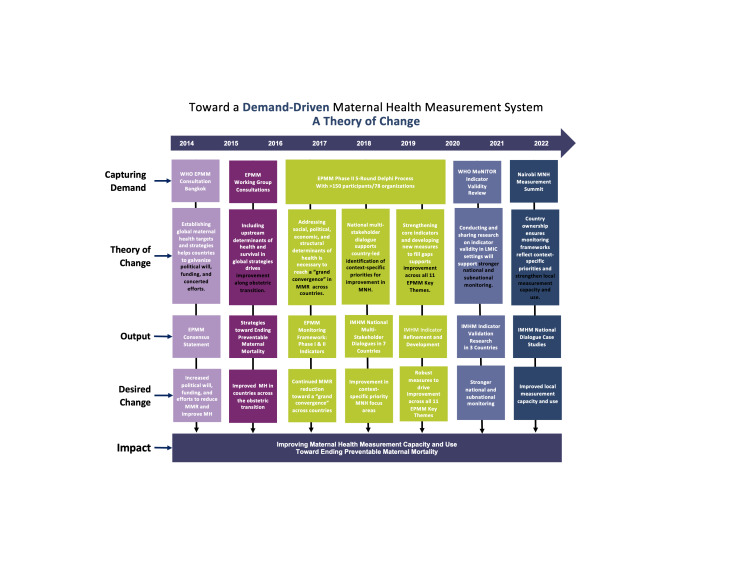
Demand-driven measurement theory of change: Ending Preventable Maternal Mortality (EPMM) – Improving Maternal Health Measurement Capacity and Use (IMHM).

This paper describes the process and outcomes of two consultations which resulted in the development of eight additional EPMM indicators, as follows:

- Consultation 1: Four indicators reflecting fundamental human rights principles applied to maternal health
- Consultation 2: Four indicators reflecting better measurement of births, deaths, and causes of death

The primary aim of these technical consultations was to affirm the identified measurement gaps, specify the critical components of the constructs for measurement, and fully elaborate metadata for these constructs (definition, numerator, denominator, disaggregators) to allow them to be monitored.

A secondary aim is to share the experience of adapting a technical consultation to an online platform due to COVID-19 travel restrictions, which may offer important lessons learned for others beyond the field of maternal health measurement.

## METHODS

### Selection of constructs for measure development

This is a qualitative, iterative, structured, remote expert technical consultation. From May 11 to July 17, 2020, the IMHM Project convened two simultaneous online technical consultations focused on developing eight additional EPMM indicators from the full list of additional measures requested to fill measurement gaps that emanated from Phase II of the EPMM indicator development process. The twenty-nine additional EPMM indicators requested (**Table 2**) were distilled into their underlying constructs. As defined by Lavrakas [9], a construct is “the abstract idea, underlying theme, or subject matter that one wishes to measure”. While the original EPMM stakeholder requests were formulated with detailed (aspirational) indicator definitions, we chose to focus at the construct level to give content experts and measure developers the freedom to deliberate on the strongest formulations of the underlying concepts of interest for the purposes of measurement.

The selection of constructs to be developed into fully articulated indicators was based on inputs collected during national multi-stakeholder dialogues in five countries, along with information from a landscape analysis based on online surveys and key informant interviews with government officials and in-country UN agency staff in twenty countries on their perceived need for the proposed additional indicators. From 2018-2020, the IMHM Project supported seven national multi-stakeholder dialogues, co-sponsored with government officials in Ministries of Health and civil society organizations and focused on collecting stakeholder opinions on the priorities for ending preventable maternal mortality and maternal health measurement needs to support their achievement in each context. At dialogues held in five of the seven countries, Kenya, Cote d’Ivoire, India, Bangladesh, Pakistan and Mexico [10], participants took part in interactive prioritization exercises in which they ranked the importance of all twenty-nine proposed additional EPMM indicators in their context, using a four-point scale. The constructs that a majority of stakeholders across all participating countries rated “Very Important” were considered for inclusion in these consultations. These results provided additional inputs into national demand for development of the proposed additional indicators.

The final selection was made with the input of a global advisory committee including maternal newborn health and measurement experts whose organizations contributed to the development of the EPMM Strategies report and its comprehensive monitoring framework. The candidate constructs were selected from the list of those deemed of importance at country level based on the following predetermined criteria:

- The construct is conceptually clear
- The construct needs measurement
- Measurement of this construct is relevant and important
- Measurement data on this construct would be actionable
- Measurement of this construct is conducive to global monitoring and national comparisons
- There are available data of acceptable quality on which to base or compile an indicator to measure this construct

Crucial to prioritization was careful consideration of indicator development already in progress by other researchers and UN agency groups, to coordinate indicator development efforts and ensure that the constructs prioritized are not duplicative of ongoing initiatives by other organizations.

In the end, eight constructs were prioritized for development into fully articulated indicators through the current consultations (**Table 3**): Consultation 1 - four rights-based constructs for measurement (construct IDs: 1.1; 1.2; 4.1; 4.2); Consultation 2 - four constructs for strengthening measurement of births and deaths (construct IDs 2.2; 5.1; 5.2; 5.4).

**Table 3:** Constructs for consideration and development into fully articulated indicators

EPMM Theme	Construct ID	Constructs for development	Original EPMM request
**1**	1.1	An indicator that measures women's decision-making power about timing and number of births	Proportion of women aged 15-49 who make their own informed and empowered decisions regarding sexual relations, contraceptive use, and reproductive health care, and the timing and number of births
	1.2	An indicator that holds local and district governments accountable for monitoring maternal health outcomes at the community level	Number of district local governments that articulate efforts of sectors accredited in its geographic area and monitor results in each community
**2**	2.2	An indicator verifying that the national health information system links births and maternal and perinatal deaths, and includes cause of death	Presence of national information system(s) that are able to record and report data as described by ICD-PM, linking outcomes (births and deaths) to maternal and perinatal conditions, and to report annually on characteristics of births, deaths, and other vital events to produce statistics relevant to monitoring of reproductive health and mortality
**4**	4.1	An indicator verifying that the national pre-service education curriculum for maternal health workers includes standards for Respectful Maternity Care (RMC)	Presence of a component that specifically addresses the Universal Rights of Childbearing Women (RMC Charter) in the national pre-service education curriculum for all midwifery service providers
	4.2	An indicator verifying that the national health plan includes the right to Respectful Maternity Care (RMC)	Presence of Respectful Maternity Care (RMC) as a right in the national health plan(s)
**5**	5.1	An indicator that tracks use of Maternal Newborn Health (MNH) data by health workers for decision making	Percentage of health workers using MNCAH data for decision-making
	5.2	An indicator that tracks coverage of death and birth registration	Death and birth registration coverage
	5.4	An indicator that tracks the capacity of the national information system to record and report maternal and newborn cause of death data	Presence of national information system(s) that are able to record and report data as described by ICD-PM

EPMM – Ending Preventable Maternal Mortality

### Consultation participants

We purposively identified experts with specific expertise in maternal health measure development as well as the topical areas covered by the constructs for development to participate. Sixty-one participants took part across both consultations. Thirty-one participants took part in Consultation 1, focused on developing fully articulated indicators for a set of Rights-Based constructs for measurement. Thirty participants took part in Consultation 2, focused on developing a set of fully articulated indicators for measuring births and deaths.

### Measure development

Due to travel restrictions imposed by the COVID-19 pandemic, measure development took place through two simultaneous, parallel ten-week online consultations stratified by focus area (see **Table 3**), hosted on Harvard Canvas, the university’s online education platform. The consultations used multiple modalities to collect inputs and conduct in-depth consultation with the convened groups of experts in order to achieve their intended objectives.

To orient participants to each construct for measurement and to affirm the identified measurement gaps, participants first watched presentations from subject matter experts on each construct for consideration. Twelve content experts gave presentations in Consultation 1. Ten content experts gave presentations in Consultation 2. Each presenter provided an overview of the current state of measurement of the construct they were presenting, as well as their own perspectives on the feasibility, challenges, and utility of measuring the construct. This set the stage and provided an introduction for the structured online discussion forums and technical work that followed for each construct to be developed into an indicator.

After watching presentations, participants completed online polls to identify the critical component(s) of each construct for measurement, choose the expression of the construct best capturing the specific measurement gap, and vote on whether to move forward to articulate the full metadata for an indicator to measure the construct. Next, following participation in a semi-structured online discussion, participants completed a final survey to consolidate the group’s consensus on which constructs to take forward. All eight constructs for measurement were ultimately ratified after considerable debate and brought forward for full indicator development, to include indicator definition and articulation of full metadata.

Participants then worked collaboratively in small groups to develop the full metadata for each construct. To prepare, each participant individually drafted their proposed formulation of all seven components of the metadata for each construct: definition, numerator, denominator, method of estimation, disaggregation factors, preferred data sources, and the expected frequency of data collection. These individual participant inputs then populated a group worksheet for each construct. In small groups on Zoom, organized by time zones, participants discussed the group worksheet for each construct, debating the merits and challenges related to the proposed formulations of each component of the metadata to reach agreement on the strongest versions of each draft indicator, using pre-specified criteria [11] (**Table 4**). In this way, participants in each small group developed consensus on a proposed draft indicator for their group, which a rapporteur submitted for consideration by the full plenary.

**Table 4:** Criteria for evaluating draft indicator metadata

**Ranking criteria**	
1. Clarity of focus and meaning	Unambiguous; reflects or represents the object of the evaluation/component accurately
2. Relevance to evaluation question/construct	Connectedness to the question/construct to be addressed
3. Comparability/consistency	Applicable in diverse settings
4. Non-directional language	Written to be neutral or without a bias, not defined as positive or negative in advance
5. Units of measurement and computational method clearly defined	Frequency, percentage, magnitude, rate, ratio, score, rate difference, trend over time, comparison to benchmark
6. Data quality	The degree to which information for this component will be complete, reliable, and valid

The proposed draft indicators for each construct from all small groups were compiled into a final poll that included one additional version of each indicator synthesized by the consultation administrators that drew from all submissions in an attempt to reflect the “best of” all the proposals. Participants then voted on the strongest draft indicator, including the definition and full metadata, for each construct.

A final plenary discussion to review these poll results was hosted on Zoom for each consultation. All participants for each consultation came together to review the full metadata for the highest-ranking draft indicator for each construct, and to discuss any related questions and concerns posted on the Canvas discussion boards. A facilitated discussion ensued to address the outstanding issues regarding any aspect of each measure, where participants arrived at agreed solutions wherever possible. The draft indicators emanating from both consultations appear in the tables below.

## RESULTS

Eight new candidate indicators with relevance for monitoring the priority recommendations highlighted in the EPMM Strategies were developed through these two simultaneous technical measure development consultations. Each indicator includes a definition, numerator and denominator (if applicable), method of estimation, disaggregation factors (stratifiers), preferred data source(s), and expected periodicity (frequency).

### Consultation 1: Four Rights-Based Indicators

Four indicators address gaps in measures to monitor fundamental rights-based determinants of maternal health and survival at national and subnational level, including women’s reproductive autonomy and decision making power; participatory accountability for maternal health outcomes [12]; and Respectful Maternity Care. These indicators address identified gaps in measures available for monitoring the following EPMM Key Themes: Theme 1. Empower women, girls, families and communities; and Theme 4. Apply a human rights framework to ensure that high-quality reproductive, maternal, and newborn health care is available, accessible and acceptable to all who need it (**Table 5**).

**Table 5:** Full metadata for four rights-based EPMM indicators (developed during Consultation 1)

	Construct 1.1	Construct 1.2	Construct 4.1	Construct 4.2
**Definition**	Proportion of women of reproductive age who make their own decisions about if, when, and how many children they want	Percentage of district governments that have established mechanisms for collection, review and response to community-led monitoring of maternal health outcomes	Percentage of health facility staff and frontline maternity care providers demonstrating knowledge, competencies, skills and behaviors standards set out by the RMC Charter	Number of countries with laws and regulations that include all ten articles of the Respectful Maternity Care Charter as rights, and require periodic monitoring, review, and reporting of RMC at national and sub-national levels
**Numerator (if applicable)**	Number of women of reproductive age who reply “Yes” to all four questions: I was able to freely decide (alone or jointly with my partner/husband): 1) whether or not to conceive a child 2) whether or not to terminate a conception 3) when to have a child 4) the number of children to have	Number of district/subnational governments with functional accountability mechanisms for collection, review and response to community-led monitoring of maternal health outcomes	Number of frontline service providers (doctors, nurses, midwives, trainees, and facility staff) who care for people during the pregnancy, childbirth, and postpartum, and newborn phases demonstrating RMC knowledge, competencies, skills and behaviors	Number of countries that answer “Yes” to the following questions: 1) Is RMC included as a right in national laws and regulations? 2) Are all ten RMC rights included as a right in laws? 3) Is there monitoring, review and reporting through participatory processes of all 10 RMC rights? 4) Is it enforceable and justiciable?
**Denominator (if applicable)**	Women of reproductive age	• Total number of district governments • Sub-indicator denominator of districts with established accountability mechanisms	All front-line service providers (doctors, nurses, midwives, trainees, and facility staff) offering pregnancy, childbirth and newborn health services, working full time or part time	Total number of countries
**Method of estimation (computation)**	• Proportion of women answering “Yes” to all four questions (%) • Score out of four components (%)	• Main indicator: Percentage of districts with mechanisms divided by total number of districts • Sub-indicator: Percentage of districts with functional mechanisms divided by districts with mechanisms • Scoring for operational/functionality levels of collection, review, and response	Scorecards and percentages for each of four components: • Percentage (%) with RMC Knowledge: Score on certification and re-certification exam (eg, >80% exam score) • Percentage (%) demonstrating acquisition of >80% RMC Competencies: Score on online and in-person training simulations • Percentage (%) demonstrating RMC Skills: Score on direct observation at facility level • Percentage (%) for RMC Practices or Behaviors: Exit interviews with women who accessed services using validated scale scores (MADM, MORI) • Summary score (presence of all four: Knowledge, Competencies, Skills, Practices/Behaviors)	Numerical or qualitative score of responses to yes/no questions
**Disaggregation factors**	• By age, marital status/duration of marriage, parity, residence, education, wealth index • By grounds of discrimination recognized in international human rights law	• By components of the indicator (collection, review, response) • By type of mechanism for each component • By local or district government	By provider cadre, in-service/pre-service, facility type, region/administrative unit, geography: rural/urban, socioeconomic status, age, parity, sex, income, ethnicity	• By each of the rights in the RMC Charter • By components of the indicator • By administrative level (national and sub-national)
**Preferred data sources**	Household or facility survey	• District health sector information reports • Community monitoring reports (civil society audits, score cards) • UN Agencies/Other partners (cross check)	Multiple sources: Facility assessments, Direct observation, OSCE evaluations, National (re)certification exams, Surveys with women	• For national monitoring: Ministry of Health reports • For global monitoring: eg, WHO Global Health Policy Database; UN Treaty Body or SDG reports
**Expected frequency of data collection**	3-5 years	Annual and 5-year aggregate reporting	Routine data collection with annual reporting	Annual

EPMM – Ending Preventable Maternal Mortality

### Consultation 2: Indicators for Measuring Births and Deaths

Four indicators strengthen the measurement of and ability to count, track, and link births and maternal deaths and causes of death. They address identified gaps in measures available for monitoring the following EPMM Key Themes: Theme 2. Integrate maternal and newborn care, protect and support the mother-baby dyad; and Theme 5. Improve metrics, measurement systems, and data quality (**Table 6**).

**Table 6:** Full metadata for four EPMM indicators for tracking births and maternal deaths (developed during Consultation 2)

	Construct 5.1	Construct 5.2	Construct 5.4	Construct 2.2
**Definition**	Proportion of districts or sub-national units with documented evidence that district health management teams reviewed MNH data during their annual workplan development process, and took data-informed actions or decisions for improving availability and quality of MNH care	• Proportion of live births registered in CRVS within one year of birth • Proportion of still births registered in CRVS within one year of delivery • Proportion of neonatal deaths registered in CRVS within one year of death • Proportion of maternal deaths registered in CRVS within 1 year of death	Presence of a national system that captures maternal and neonatal deaths and their causes and stillbirths according to an existing international standard classification system	Capacity of a national system for civil registration of vital statistics (CRVS) and a national health information system (NHIS) to effectively link health individual level data and cover births, maternal and child deaths, and cause of death information
**Numerator (if applicable)**	Number of districts or sub-national units with: 1) documented evidence of review of health system data 2) costed action plans in their annual workplan to address issues of MNH availability and quality of care identified based on MNH health facility data	• Number of live births registered in CRVS within 1 year of birth • Number of stillbirths registered in CRVS within 1 year of delivery • Number of neonatal deaths registered in CRVS within 1 year of death • Number of maternal deaths registered in CRVS within 1 year of death	Score that captures: • Deaths registered: maternal, newborn, stillbirths (5.2) • Cause of death assigned: maternal, newborn, stillbirths (of those, % with COD) • International classification used for cause of death (of those % correctly classified) • Capture of deaths across settings: public, private, community deaths/stillbirths • System in place to assess quality of data: completeness, timeliness of reporting • Data are publicly available • Data are reported	• Presence of digital birth record (most likely CRVS) • Standard set of variables included for birth records (maternal age, congenital defects, birthweight, gestational age, etc.) • Presence of digital death certificate (most likely CRVS) • Standard set of variables included in death records (eg, timing, location, cause) • A) Presence of unique national identifier in both birth and death records to allow linkages, or • B) Standard set of variables included in birth and death records to allow for linking birth and death records • Stillbirths captured in both births and deaths
**Denominator (if applicable)**	Total number of districts or sub-national units	• Total live births (CBR, projections) • Total still births (projections, estimates) • Total neonatal deaths (projections/estimation) • Total maternal deaths (estimation)	N/A	N/A
**Method of estimation (computation)**	Percent of districts with 1 & 2	Percentage	Score: x pts / x total possible	Score
**Disaggregation factors**	• By indicator components • By region; urban/rural • By type of facility • By type of care/service; whether maternal or newborn data	By place of event (facility birth or death/home birth or death); national administrative/geographical units; by outcomes (stillbirth rate, neonatal mortality rate, maternal mortality rate)	• By type of death: maternal, newborn, stillbirths • By timing of stillbirth: antepartum, intrapartum • By subnational unit: region/state	By indicator components to know which aspect needs attention
**Preferred Data Sources**	District level reporting of: • Records from management team data review meetings • Costed workplans	• Numerator: CRVS • Denominator: gold standard/best available from multiple data sources (like surveys, census projections, mathematical models) for estimation	National report with some form of verification (eg, WHO Policy Survey)	Self-administered survey of CRVS; could also be integrated into WHO Policy Survey
**Expected Frequency of data collection**	• Monthly or quarterly for evidence of data review meetings • Annual reporting	Annual	None specified	1-2 years

CRVS – civil registration and vital statistics, COD – cause of death, EPMM – Ending Preventable Maternal Mortality

## DISCUSSION

### Implications in context of existing research

The proposed indicators contribute toward a comprehensive monitoring framework for the EPMM Strategies. As such, each proposed indicator corresponds to a specific EPMM Key Theme, and aims to fill gaps identified by stakeholders who were asked to evaluate existing maternal health metrics using pre-determined criteria to the strongest available indicators to track progress toward achieving the priority recommendations in the area of each EPMM Key Theme.

A recent commentary on measurement criticized the siloed, top-down nature of measure development, which for too long has remained an internally facing activity, controlled largely by what the authors call a “global measurement enterprise”, lacking inclusivity and transparency [13]. In contrast, the draft indicators presented in this paper reflect the results of a process driven by end-user and country-level stakeholder demand generated through a participatory process, and include the inputs and aspirations of numerous stakeholders, gathered over time and across various platforms.

These draft indicators respond to the call for a broadened approach to measurement and for indicators useful for tracking the societal determinants and health system drivers of maternal health, in addition to those that track coverage of clinical interventions and their associated outcomes that, while critical, are not sufficient [6]. Indicators that permit countries to track rights-based constructs such as women’s reproductive autonomy and decision-making power, participatory mechanisms for driving subnational accountability for maternal health outcomes, and the legal and regulatory frameworks to support Respectful Maternity Care are also needed. Such indicators provide a basis for monitoring the enabling environment for high-quality maternal newborn health care and a high-performing maternity care system. This enabling environment includes the promotion and protection of fundamental rights, attention to power dynamics, information systems, and organizational and sociopolitical dynamics.

A number of these indicators respond to the need to strengthen civil registration and vital statistics [CRVS] systems in many low- and middle-income countries, which are critical mechanisms to address and ensure citizen rights, improve gender equality, and enable health system planning and improvement [14]. Indicators that track cross-system design and interoperability and monitor national health information and registration system capacity–including the ability to capture health data and link them with data on vital events, coverage of birth and death registration, and the use of CRVS information not only for the purposes of ensuring legal status but as data for decision making–provide opportunities to improve measurement, metrics, and data quality in the context of maternal health and survival [2].

### Strengths and limitations

The iterative, discursive exploration of the concepts for measurement and the need for metrics to track them responds to recent calls for measure development to be carried out in more inclusive ways and to include a broader range of stakeholder perspectives [13]. The process undertaken to identify measurement gaps through the EPMM indicator development consultations (2016-2017), and to prioritize the constructs for measure development through national dialogues and key informant interviews in over 20 countries (2018-2019) aligns well with a recent update and reaffirmation of the Kirkland principles for maternal health measurement, calling for measure development to be country-driven with supportive global leadership, focused for purpose, relevant to the end users, innovative in design, and sensitive to measurement of equity [5].

Another strength is the focus on concept-driven measure development. Recent work to improve the understanding of and the approaches to determining indicator validity in the context of maternal and newborn health measurement have focused on the critical importance of construct validity. In a definitional framework commissioned by Mother Newborn Information for Tracking Outcomes and Results (MoNITOR) [15], an expert advisory group to the WHO on maternal newborn health measurement [16], describe construct validity as the degree to which “a given operationalization (through indicator definition and its measurement) accurately reflects the phenomenon it is intended to measure.” This essential aspect of validity was highly valued by practitioners in the field who contributed to a landscape analysis by the same authors [17] that informed the MoNITOR definitional framework. Policy dialogue on measure development emphasizes that it should be driven by a conceptual need and framing, rather than by what data are available or what is currently feasible to measure [18]]. In response, the process to develop these additional EPMM indicators focused heavily on careful consideration of the underlying construct for measurement and consensus on the best expression of that construct in each case.

The virtual format of the consultation, paired with the COVID-19 pandemic and differing time zones in which the participants completed activities, led to a few limitations. The online Harvard Canvas platform posed difficulties for a small number of participants due to occasional technical glitches, poor internet connection, or user error. Additionally, participant time zones ranged from UTC/GMT – 8 hours to UTC/GMT + 6 hours, which made it difficult for all participants to participate in all activities. Finally, although the online consultations comprised the same number of hours as the planned in-person meetings, simply spread out across a longer time frame, “consultation fatigue” over the ten-week period may have led to lower rates of response to polls or participation in other activities compared to an in-person format held over four consecutive days. The impact of the COVID-19 pandemic, which achieved global magnitude just prior to the start of the consultations, on participants work and family lives must also be recognized.

On the other hand, the online format offered numerous benefits. Although Zoom meetings and online discussion boards are not a replacement for in-person collaboration, Canvas allowed all objectives of the consultation to be met during a global pandemic. The extended time frame allowed for more in-depth analysis of each construct, including rich discussions and participant polls during each step of the consultation. Additionally, the online format allowed participants to return to past objectives and review content at their leisure, such as the recorded speaker presentations and the background resources. Furthermore, the online format allowed six more participants to take part in the consultation than could have participated in person, and enabled a variety of activities tailored to each objective, including videos, discussion forums, polls, and live Zoom meetings. In the end, 83% of those who responded to the evaluations for both consultations strongly or very strongly agreed that the consultations were effective in meeting their objectives and that they learned a lot, while 67% said they would recommend this type of online consultation to others.

## CONCLUSIONS

While recognizing that further technical and policy work remains before these draft indicators can be broadly recommended for national and subnational level monitoring and some global comparisons, they represent a solid foundation for such next steps. The inputs that went into the selection of these indicators, gathered over time, across various geographies, and from multiple stakeholder groups, ensure that the need and demand for these measures is real. The thoughtful deliberations collected during these consultations ensure that the constructs for measurement are well-defined, and that the proposed draft indicators and metadata to allow their measurement reflect the expertise of a sizeable number of measure developers and subject matter experts. Together, these indicators contribute to country capacity to improve maternal health outcomes by analyzing and acting on influential social and health system level determinants of maternal health and survival [19].
